# Supplementary material for: Hot spot prediction in protein-protein interactions by an ensemble system
Source: BMC Syst Biol. 2018 Dec 31;12(Suppl 9):132. doi: 10.1186/s12918-018-0665-8 (PMC6311905; doi:10.1186/s12918-018-0665-8)
Supplement: Supplementary file 2 — All relevant MATLAB code. A simple Matlab implement of our predictor. (ZIP 19,742 kb) [file 12918_2018_665_MOESM2_ESM.zip › Additional file 2/Read ASA/ASA website/SKEMPI/1GC1C.htm]

 NetSurfP - Protein Surface Accessibility and Secondary Structure Predictions

  

|  |  |  |
| --- | --- | --- |
|  |  | NetSurfP - Protein Surface Accessibility and Secondary Structure Predictions  Technical University of Denmark |

  


---

```
# For publication of results, please cite:
# A generic method for assignment of reliability scores applied to solvent accessibility predictions.
# Bent Petersen, Thomas Nordahl Petersen, Pernille Andersen, Morten Nielsen and Claus Lundegaard
# BMC Structural Biology 2009, 9:51 doi:10.1186/1472-6807-9-51
#
# Column 1: Class assignment - B for buried or E for Exposed - Threshold: 25% exposure, but not based on RSA
# Column 2: Amino acid
# Column 3: Sequence name
# Column 4: Amino acid number
# Column 5: Relative Surface Accessibility - RSA
# Column 6: Absolute Surface Accessibility
# Column 7: Z-fit score for RSA prediction
# Column 8: Probability for Alpha-Helix
# Column 9: Probability for Beta-strand
# Column 10: Probability for Coil
E K  1GC1C                  1    0.748 153.925   1.364   0.003   0.003   0.994
E K  1GC1C                  2    0.626 128.686   0.782   0.004   0.420   0.576
E V  1GC1C                  3    0.428  65.753   1.323   0.001   0.900   0.099
B V  1GC1C                  4    0.110  16.830   0.650   0.001   0.959   0.040
E L  1GC1C                  5    0.404  73.936   1.586   0.000   0.983   0.017
B G  1GC1C                  6    0.068   5.383  -0.162   0.001   0.959   0.040
E K  1GC1C                  7    0.463  95.177   1.733   0.001   0.959   0.040
E K  1GC1C                  8    0.427  87.752   1.001   0.003   0.718   0.279
E G  1GC1C                  9    0.646  50.832  -0.492   0.004   0.085   0.910
E D  1GC1C                 10    0.550  79.313   1.233   0.004   0.138   0.858
E T  1GC1C                 11    0.531  73.664   1.414   0.003   0.718   0.279
B V  1GC1C                 12    0.044   6.809   0.328   0.001   0.959   0.040
E E  1GC1C                 13    0.364  63.678   1.883   0.001   0.959   0.040
B L  1GC1C                 14    0.045   8.258   0.510   0.001   0.959   0.040
E T  1GC1C                 15    0.364  50.556   1.429   0.001   0.959   0.040
B C  1GC1C                 16    0.025   3.552   0.441   0.001   0.900   0.099
E T  1GC1C                 17    0.442  61.319   1.262   0.001   0.900   0.099
B A  1GC1C                 18    0.071   7.824  -2.101   0.001   0.900   0.099
E S  1GC1C                 19    0.583  68.292   0.235   0.004   0.514   0.481
E Q  1GC1C                 20    0.710 126.824  -1.814   0.004   0.085   0.910
E K  1GC1C                 21    0.480  98.736  -0.493   0.005   0.015   0.979
E K  1GC1C                 22    0.609 125.189  -1.116   0.005   0.045   0.951
E S  1GC1C                 23    0.575  67.367   0.546   0.004   0.138   0.858
B I  1GC1C                 24    0.122  22.589   0.381   0.004   0.420   0.576
E Q  1GC1C                 25    0.388  69.261   1.334   0.001   0.900   0.099
B F  1GC1C                 26    0.036   7.205  -0.101   0.001   0.959   0.040
E H  1GC1C                 27    0.260  47.258   1.399   0.001   0.959   0.040
B W  1GC1C                 28    0.036   8.562  -0.123   0.011   0.918   0.071
E K  1GC1C                 29    0.311  63.993   1.040   0.001   0.959   0.040
B N  1GC1C                 30    0.209  30.554   0.884   0.003   0.718   0.279
E S  1GC1C                 31    0.611  71.586  -0.765   0.005   0.045   0.951
E N  1GC1C                 32    0.746 109.214  -1.463   0.005   0.015   0.979
E Q  1GC1C                 33    0.541  96.712  -1.083   0.004   0.420   0.576
E I  1GC1C                 34    0.505  93.407  -0.487   0.003   0.718   0.279
E K  1GC1C                 35    0.343  70.452   0.173   0.021   0.756   0.223
B I  1GC1C                 36    0.241  44.548  -0.509   0.021   0.756   0.223
B L  1GC1C                 37    0.251  45.940  -1.259   0.022   0.552   0.426
E G  1GC1C                 38    0.447  35.171  -1.581   0.021   0.279   0.699
E N  1GC1C                 39    0.451  66.085  -1.743   0.019   0.141   0.840
E Q  1GC1C                 40    0.533  95.140  -1.646   0.019   0.141   0.840
E G  1GC1C                 41    0.477  37.579  -1.617   0.020   0.205   0.775
E S  1GC1C                 42    0.479  56.186  -0.580   0.023   0.655   0.322
E F  1GC1C                 43    0.266  53.346  -0.682   0.021   0.756   0.223
E L  1GC1C                 44    0.261  47.716  -0.302   0.018   0.846   0.136
E T  1GC1C                 45    0.374  51.888  -1.027   0.023   0.655   0.322
E K  1GC1C                 46    0.540 110.996  -0.877   0.021   0.451   0.528
E G  1GC1C                 47    0.498  39.177  -1.752   0.020   0.205   0.775
E P  1GC1C                 48    0.536  76.016  -1.018   0.064   0.216   0.721
E S  1GC1C                 49    0.495  58.049  -1.297   0.066   0.296   0.638
E K  1GC1C                 50    0.536 110.276  -0.573   0.066   0.296   0.638
E L  1GC1C                 51    0.340  62.181  -1.168   0.066   0.296   0.638
E N  1GC1C                 52    0.477  69.877  -1.402   0.064   0.216   0.721
E D  1GC1C                 53    0.479  69.053  -1.759   0.021   0.279   0.699
E R  1GC1C                 54    0.348  79.692  -0.911   0.021   0.451   0.528
B A  1GC1C                 55    0.312  34.327  -1.610   0.022   0.552   0.426
E D  1GC1C                 56    0.490  70.652  -1.161   0.022   0.552   0.426
B S  1GC1C                 57    0.287  33.625  -1.402   0.021   0.451   0.528
E R  1GC1C                 58    0.446 102.111  -1.025   0.021   0.451   0.528
E R  1GC1C                 59    0.484 110.928  -0.983   0.074   0.484   0.442
E S  1GC1C                 60    0.467  54.721  -1.126   0.022   0.359   0.619
E L  1GC1C                 61    0.387  70.951  -1.178   0.022   0.359   0.619
E W  1GC1C                 62    0.397  95.527  -1.353   0.021   0.451   0.528
E D  1GC1C                 63    0.560  80.739  -1.494   0.022   0.359   0.619
E Q  1GC1C                 64    0.511  91.211  -1.631   0.004   0.138   0.858
E G  1GC1C                 65    0.541  42.545  -2.001   0.004   0.085   0.910
E N  1GC1C                 66    0.541  79.173  -0.731   0.004   0.197   0.799
B F  1GC1C                 67    0.109  21.836  -0.546   0.004   0.514   0.481
E P  1GC1C                 68    0.233  32.992   0.676   0.002   0.816   0.182
B L  1GC1C                 69    0.021   3.827   0.867   0.001   0.959   0.040
E I  1GC1C                 70    0.245  45.399   1.384   0.001   0.959   0.040
B I  1GC1C                 71    0.029   5.365   0.157   0.001   0.900   0.099
E K  1GC1C                 72    0.446  91.763   1.437   0.002   0.816   0.182
E N  1GC1C                 73    0.529  77.475   0.264   0.004   0.514   0.481
B L  1GC1C                 74    0.129  23.602   0.286   0.005   0.262   0.733
E K  1GC1C                 75    0.505 103.837   0.956   0.005   0.336   0.660
E I  1GC1C                 76    0.537  99.364   0.050   0.005   0.336   0.660
E E  1GC1C                 77    0.654 114.306   0.123   0.004   0.138   0.858
B D  1GC1C                 78    0.238  34.267  -0.844   0.004   0.085   0.910
E S  1GC1C                 79    0.551  64.589  -0.238   0.004   0.138   0.858
B D  1GC1C                 80    0.216  31.082  -1.211   0.003   0.718   0.279
E T  1GC1C                 81    0.428  59.391   1.872   0.001   0.900   0.099
B Y  1GC1C                 82    0.061  12.972   0.669   0.001   0.959   0.040
E I  1GC1C                 83    0.280  51.818   2.062   0.001   0.959   0.040
B C  1GC1C                 84    0.018   2.457   1.240   0.001   0.959   0.040
E E  1GC1C                 85    0.297  51.938   1.877   0.001   0.959   0.040
B V  1GC1C                 86    0.027   4.135   0.074   0.001   0.959   0.040
E E  1GC1C                 87    0.489  85.428   0.690   0.002   0.816   0.182
E D  1GC1C                 88    0.389  56.098  -0.410   0.004   0.420   0.576
E Q  1GC1C                 89    0.610 108.928  -0.239   0.004   0.138   0.858
E K  1GC1C                 90    0.617 126.835  -0.109   0.005   0.336   0.660
E E  1GC1C                 91    0.459  80.222  -0.426   0.003   0.718   0.279
E E  1GC1C                 92    0.473  82.598   0.706   0.001   0.900   0.099
B V  1GC1C                 93    0.141  21.702  -0.190   0.001   0.900   0.099
E Q  1GC1C                 94    0.398  71.047   0.495   0.001   0.900   0.099
B L  1GC1C                 95    0.120  21.972  -0.197   0.001   0.900   0.099
B L  1GC1C                 96    0.270  49.419   0.783   0.001   0.900   0.099
B V  1GC1C                 97    0.208  31.908  -0.071   0.001   0.900   0.099
B F  1GC1C                 98    0.229  45.920   0.422   0.001   0.900   0.099
E G  1GC1C                 99    0.550  43.277   0.414   0.001   0.900   0.099
B L  1GC1C                100    0.103  18.841   0.235   0.002   0.816   0.182
E T  1GC1C                101    0.582  80.779   0.096   0.004   0.616   0.381
E A  1GC1C                102    0.277  30.503   0.085   0.005   0.262   0.733
E N  1GC1C                103    0.481  70.433  -0.167   0.004   0.085   0.910
E S  1GC1C                104    0.513  60.147  -1.182   0.005   0.045   0.951
E D  1GC1C                105    0.682  98.276   0.204   0.004   0.138   0.858
B T  1GC1C                106    0.225  31.235   0.626   0.004   0.616   0.381
E H  1GC1C                107    0.538  97.880   0.986   0.001   0.900   0.099
B L  1GC1C                108    0.122  22.265   0.421   0.001   0.900   0.099
E L  1GC1C                109    0.438  80.198   1.219   0.002   0.816   0.182
E Q  1GC1C                110    0.517  92.372   0.499   0.005   0.262   0.733
E G  1GC1C                111    0.650  51.116  -0.833   0.005   0.015   0.979
E Q  1GC1C                112    0.448  79.941   0.812   0.005   0.045   0.951
E S  1GC1C                113    0.530  62.116   1.107   0.004   0.514   0.481
B L  1GC1C                114    0.049   8.954   0.683   0.001   0.900   0.099
E T  1GC1C                115    0.398  55.258   2.081   0.001   0.959   0.040
B L  1GC1C                116    0.054   9.851   0.951   0.001   0.959   0.040
E T  1GC1C                117    0.369  51.166   1.882   0.001   0.959   0.040
B L  1GC1C                118    0.022   4.101   0.703   0.001   0.959   0.040
E E  1GC1C                119    0.494  86.302   1.303   0.001   0.900   0.099
B S  1GC1C                120    0.070   8.169  -1.592   0.002   0.816   0.182
E P  1GC1C                121    0.601  85.339   0.241   0.004   0.514   0.481
E P  1GC1C                122    0.515  73.022  -1.993   0.004   0.138   0.858
E G  1GC1C                123    0.570  44.828  -0.562   0.004   0.085   0.910
B S  1GC1C                124    0.240  28.128  -0.541   0.005   0.045   0.951
E S  1GC1C                125    0.537  62.972   0.866   0.004   0.138   0.858
B P  1GC1C                126    0.098  13.935  -0.074   0.004   0.514   0.481
E S  1GC1C                127    0.415  48.615   1.194   0.001   0.900   0.099
B V  1GC1C                128    0.044   6.778   0.479   0.001   0.959   0.040
E Q  1GC1C                129    0.316  56.402   1.609   0.001   0.959   0.040
B C  1GC1C                130    0.033   4.647   0.112   0.001   0.959   0.040
E R  1GC1C                131    0.326  74.768   1.159   0.001   0.900   0.099
B S  1GC1C                132    0.210  24.600   0.999   0.004   0.616   0.381
E P  1GC1C                133    0.675  95.768  -0.114   0.005   0.015   0.979
E R  1GC1C                134    0.773 177.040  -1.534   0.003   0.003   0.994
E G  1GC1C                135    0.606  47.716  -1.068   0.005   0.336   0.660
E K  1GC1C                136    0.599 123.297   0.204   0.004   0.616   0.381
E N  1GC1C                137    0.341  49.922   0.053   0.023   0.655   0.322
E I  1GC1C                138    0.379  70.189   0.293   0.004   0.514   0.481
E Q  1GC1C                139    0.570 101.856  -0.794   0.005   0.262   0.733
E G  1GC1C                140    0.777  61.150  -0.567   0.005   0.045   0.951
E G  1GC1C                141    0.704  55.397  -0.517   0.005   0.045   0.951
E K  1GC1C                142    0.403  82.815   0.306   0.005   0.262   0.733
E T  1GC1C                143    0.417  57.824   1.392   0.002   0.816   0.182
B L  1GC1C                144    0.036   6.500  -0.145   0.001   0.959   0.040
E S  1GC1C                145    0.339  39.766   1.941   0.001   0.959   0.040
B V  1GC1C                146    0.047   7.209   0.554   0.001   0.959   0.040
E S  1GC1C                147    0.450  52.763   1.614   0.002   0.816   0.182
E Q  1GC1C                148    0.566 101.105   0.999   0.003   0.718   0.279
B L  1GC1C                149    0.095  17.340   0.165   0.004   0.420   0.576
E E  1GC1C                150    0.458  79.960   0.941   0.004   0.514   0.481
E L  1GC1C                151    0.518  94.827   0.278   0.004   0.420   0.576
E Q  1GC1C                152    0.589 105.160  -0.564   0.004   0.197   0.799
E D  1GC1C                153    0.286  41.241  -0.628   0.004   0.085   0.910
E S  1GC1C                154    0.486  56.959  -0.713   0.004   0.138   0.858
B G  1GC1C                155    0.157  12.395  -1.828   0.003   0.718   0.279
E T  1GC1C                156    0.409  56.756   1.806   0.001   0.900   0.099
B W  1GC1C                157    0.059  14.310   0.676   0.001   0.959   0.040
B T  1GC1C                158    0.272  37.685   1.660   0.001   0.959   0.040
B C  1GC1C                159    0.017   2.387   1.220   0.001   0.959   0.040
E T  1GC1C                160    0.257  35.646   1.877   0.001   0.959   0.040
B V  1GC1C                161    0.039   5.979  -0.455   0.001   0.959   0.040
E L  1GC1C                162    0.387  70.951   0.396   0.001   0.900   0.099
B Q  1GC1C                163    0.160  28.558   0.041   0.004   0.420   0.576
E N  1GC1C                164    0.754 110.386   0.316   0.005   0.045   0.951
E Q  1GC1C                165    0.515  92.015  -0.795   0.004   0.138   0.858
E K  1GC1C                166    0.528 108.548  -0.733   0.004   0.420   0.576
E K  1GC1C                167    0.494 101.698  -0.242   0.002   0.816   0.182
E V  1GC1C                168    0.319  48.969  -0.001   0.001   0.959   0.040
E E  1GC1C                169    0.485  84.799   0.337   0.001   0.959   0.040
B F  1GC1C                170    0.195  39.096   0.774   0.001   0.959   0.040
E K  1GC1C                171    0.423  86.929   0.712   0.001   0.959   0.040
B I  1GC1C                172    0.049   9.028   0.411   0.001   0.959   0.040
E D  1GC1C                173    0.376  54.153   1.670   0.001   0.959   0.040
B I  1GC1C                174    0.031   5.661   0.327   0.001   0.959   0.040
E V  1GC1C                175    0.263  40.362   1.813   0.001   0.959   0.040
B V  1GC1C                176    0.052   7.916   0.612   0.001   0.959   0.040
E L  1GC1C                177    0.246  45.134   1.288   0.001   0.959   0.040
B A  1GC1C                178    0.192  21.125  -1.288   0.002   0.816   0.182
E F  1GC1C                179    0.259  51.981   0.461   0.004   0.420   0.576
E Q  1GC1C                180    0.505  90.122   0.258   0.004   0.197   0.799
E K  1GC1C                181    0.836 171.945   0.858   0.016   0.005   0.979

Explain the output.  Go back.
```

---
